# The feasibility of nurse practitioner-performed, telementored lung telesonography with remote physician guidance - ‘a remote virtual mentor’

**DOI:** 10.1186/2036-7902-5-5

**Published:** 2013-06-27

**Authors:** Nancy Biegler, Paul B McBeth, Corina Tiruta, Douglas R Hamilton, Zhengwen Xiao, Innes Crawford, Martha Tevez-Molina, Nat Miletic, Chad G Ball, Linping Pian, Andrew W Kirkpatrick

**Affiliations:** 1 Regional Trauma Services, Foothills Medical Centre, Calgary, Alberta T2N 2T9, Canada; 2 Department of Surgery, Foothills Medical Centre, Calgary, Alberta T2N 2T9, Canada; 3 Department of Medicine, Foothills Medical Centre, Calgary, Alberta T2N 2T9, Canada; 4 Department of Critical Care Medicine, Foothills Medical Centre, Calgary, Alberta T2N 2T9, Canada; 5 University of Aberdeen, Aberdeen, AB24 3UE, Scotland; 6 Patient Care Systems, Alberta Health Services, Edmonton, Alberta T5J 3E4, Canada; 7 The First Affiliated Hospital of Henan University of Traditional Chinese Medicine, Zhengzhou, Henan 450000, China; 8 The Snyder Institute for Chronic Diseases, Calgary, Alberta T2N 4N1, Canada; 9 Canadian Forces Health Services, Ottawa, Ontario K1A 0K2, Canada

**Keywords:** Pneumothorax, Remote medicine, Tele-ultrasound, Education, Global health

## Abstract

**Background:**

Point-of-care ultrasound (POC-US) use is increasingly common as equipment costs decrease and availability increases. Despite the utility of POC-US in trained hands, there are many situations wherein patients could benefit from the added safety of POC-US guidance, yet trained users are unavailable. We therefore hypothesized that currently available and economic ‘off-the-shelf’ technologies could facilitate remote mentoring of a nurse practitioner (NP) to assess for recurrent pneumothoraces (PTXs) after chest tube removal.

**Methods:**

The simple remote telementored ultrasound system consisted of a handheld ultrasound machine, head-mounted video camera, microphone, and software on a laptop computer. The video output of the handheld ultrasound machine and a macroscopic view of the NP's hands were displayed to a remote trauma surgeon mentor. The mentor instructed the NP on probe position and US machine settings and provided real-time guidance and image interpretation via encrypted video conferencing software using an Internet service provider. Thirteen pleural exams after chest tube removal were conducted.

**Results:**

Thirteen patients (26 lung fields) were examined. The remote exam was possible in all cases with good connectivity including one trans-Atlantic interpretation. Compared to the subsequent upright chest radiograph, there were 4 true-positive remotely diagnosed PTXs, 2 false-negative diagnoses, and 20 true-negative diagnoses for 66% sensitivity, 100% specificity, and 92% accuracy for remotely guided chest examination.

**Conclusions:**

Remotely guiding a NP to perform thoracic ultrasound examinations after tube thoracostomy removal can be simply and effectively performed over encrypted commercial software using low-cost hardware. As informatics constantly improves, mentored remote examinations may further empower clinical care providers in austere settings.

## Background

Ultrasound is a portable, non-ionizing diagnostic tool that can instantly augment the information available to a bedside clinician. Its use at the bedside is increasing every day as ultrasound machines and technique improve [1]. Accordingly, the World Health Organization has considered ultrasound to be one of the most important technologies developing countries need, rating access to general-purpose ultrasonography as a minimum global standard [2]. Ultrasound, however, is typically very user dependent, meaning the technology may often be available, but trained users are not. It is therefore important to develop techniques to compensate for this.

Remote telementored ultrasound (RTMUS) consists of the use of informatics technologies to facilitate the real-time guidance by a remote expert of an on-site less-experienced ultrasound user to generate and interpret in real-time, meaningful images that guide the care of the patient [3]. This paradigm was initiated and pioneered by investigators from the National Aeronautics and Space Administration as the only practical solution to provide emergency medical imaging onboard the International Space Station [4]. In these studies, novice non-medical users were guided from the ground to generate diagnostic quality images of multiple body regions by ultrasound experts. A spin-off result of this approach included supporting examiners of simulated patients in a variety of test settings [5,6]. Taking advantage of the informatics capabilities of handheld smartphones has led to the demonstration that these devices can themselves be used to facilitate RTMUS [7].

While these initial demonstrations suggest that RTMUS may be practical whenever an ultrasound can access the Internet, experience in true clinical encounters is limited. We thus sought to advance the understanding of how RTMUS might empower clinical care by evaluating the ability to remotely mentor non-physician caregivers, namely nurses, to examine the pleural interface for evidence of pneumothorax (PTX) in patients after tube thoracostomy (TT) removal.

## Methods

The Foothills Medical Centre is a quaternary care, Trauma Association of Canada-accredited level I regional trauma center and the largest hospital in Alberta, Canada, providing advanced health care services to over two million people. Multi-system trauma patients, of whom over 1,000 ISS > 12 are admitted yearly, are cared for using a multi-disciplinary team approach with the team led by an attending trauma surgeon who supervises and directs all phases of care through a collaborative model. Patients who have had a TT placed for any post-traumatic reason are followed daily by the attending trauma team who decides upon the appropriate time for TT removal based on consideration of any and/or all of the following: clinical condition and anticipated course, necessity for pleural suction, pleural drainage of fluid/air, and daily chest radiograph (CXR) results. Although the attending surgeon determines the suitability of TT removal, the physical removal is conducted by the patient's assigned nurse both in the intensive care unit (ICU) and on the trauma ward. CXRs are obtained soon after removal to rule out post-TT removal complications, primarily recurrent PTXs. Formal CXR reports dictated by attending radiologists are typically available hours to days later, with the dictated CXR report being considered the ‘reference standard’ test for statistical comparison. Patients were recruited on a convenience basis by the nurse practitioner based on appropriate patients requiring TT removal and examiner availability. This study was approved for waiver of consent by the Conjoint Ethics Boards. Further, no distinguishable patient characteristics or images were reported in the manuscript.

### Equipment

A RTMUS system was composed of easily accessible off-the-shelf hardware:

• The National Television System Committee analog video output from a NanoMaxx™ ultrasound (NanoMaxx, Sonosite Corporation, Bothell, WA, USA) was digitized using a USB video CODEC (Monoprice, Rancho Cucamonga, CA, USA).

• A head-mounted 1.3-megapixel web camera (Microsoft, Redmond, WA, USA) provided a regional view of the examination, allowing the remote examiner to view both the patient and examiner's hands.

• The abovementioned images were combined with custom-designed graphical user interface (GUI) software that itself used the freely available software XSplit Broadcaster (SplitMediaLabs Ltd., Hong Kong) on a laptop computer (Lenovo ThinkPad, Hong Kong, China) running Windows XP (Microsoft Corporation).

• Skype (Luxembourg City, Luxembourg) was used to VOIP stream video and audio between the mentor and point-of-care ultrasound (POC-US) user. The selected window capture feature of Skype was used to transmit the video image of the GUI.

• The remote mentor and POC-US user used a combination of fixed LAN and Wi-Fi encrypted Internet connectivity under strict configuration control by hospital information services.

Using this RTMUS system, the remote mentor was able to view the patient and examiner's hands holding the ultrasound probe while simultaneously viewing the resultant ultrasound image in the same field of view displayed over Skype. Both the examiner and the mentor could verbally communicate over Skype. All sessions were conducted with the mentor using a laptop computer (Hewlett Packard ProBook 4520 s, Hewlett Packard, Mississauga, Ontario, Canada), through which the mentor could be viewed through the mentor's embedded web camera. After the initial introductions, however, the mentor's video display was deactivated to increase connectivity speed.

### Technique

All examinations were conducted after TT removal and prior (and thus blind) to CXR. All examinations were conducted over Skype, with the involved nurse initiating a video call once connectivity had been established following secure log-in. All actual examinations were conducted by a credentialed NP except for the final examination, which was conducted by the patient's assigned registered nurse. Neither nurse had any formal ultrasound training or prior ultrasound experience, while the remote mentor had extensive experience as a clinical ultrasonographer and teacher. The remote mentor was neither informed of the clinical history of the patient being examined nor of the side from which the TT had been removed.

**Figure 1:**
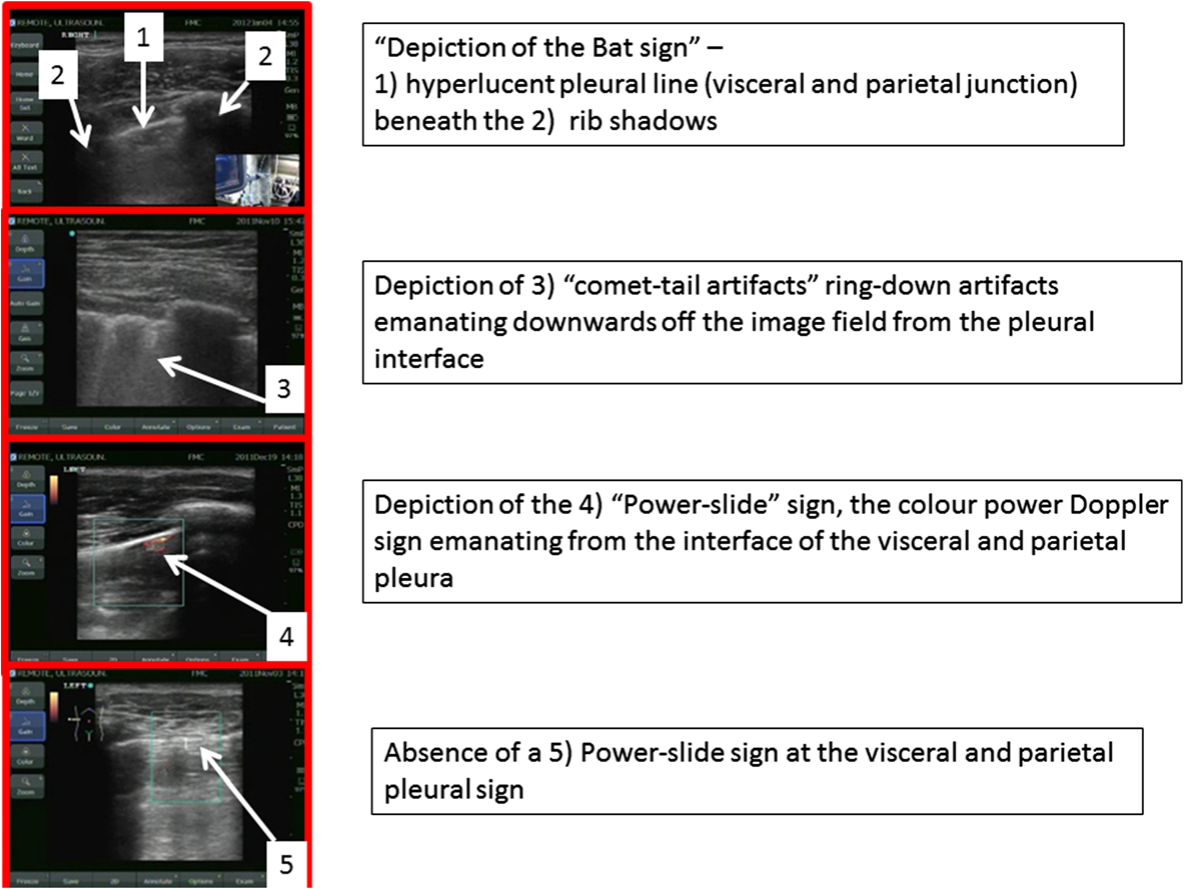
Selected ultrasound signs utilized within the WINFOCUS algorithm for pneumothorax detection.

Using lung ultrasound to exclude PTX relies on the concept that if movement of the visceral upon the parietal pleura is seen, then the two pleural surfaces are contiguous and thus cannot be separated by air. Therefore, by definition, PTX is excluded. The mentor therefore guided the nurse to manipulate the probe to demonstrate the ‘bat sign of Lichtenstein’ (Figure 1) representing the pleural interface of the visceral and the parietal pleura beneath two contiguous rib shadows [8,9]. Thereafter, the mentor and nurse collaboratively aimed to discern whether lung sliding was present, utilizing the diagnostic algorithms for pneumothorax detection promulgated by the World International Network Focused on Critical Ultrasound [10]. If present, this was documented through capturing still images demonstrating a color power Doppler signal at the pleural interface (power-slide sign) [11]. After the NanoMaxx ultrasound machine underwent a software upgrade in January 2012, allowing the additional modality of time-motion mode, the examination included documenting visible sliding through capture of a typical ‘seashore’ image using M-mode [9,12]. If typical sliding was not seen during the examination, its absence was attempted to be documented through the archiving of an absent power-slide [11,12] at the pleural interface and through capture of the ‘stratosphere sign’ [9] after the software upgrade. With absent lung sliding, the lung-point sign was also sought as a definitive sign of PTX [9,13]. Subcutaneous emphysema was also sought and defined as the presence of typical ultrasonographic air detected at an anatomic location superior to the pleural interface. Further signs sought to enable detection or exclusion of PTXs were B-lines and the lung pulse [10,14]. At the completion of each examination, the mentor and nurse discussed the totality of the findings and made and documented a clinical ‘call’ concerning the presence or absence of a post-TT removal PTX (Table 1). Formal examination times were not recorded. The study was conducted under the auspices of the Conjoint Ethics Board (Ethics IDs 17239 and 20949).

**Table 1:** Remote telementored post-tube thoracostomy removal lung examinations

**Case**	**Nurse location**	**Mentor location**	**Right lung field**	**Left lung field**	**Comment**
1	Trauma ward	Office	True negative	False negative	Very tiny apical PTX on upright CXR, clinically insignificant
2	Trauma ward	Office	True negative	True positive	PTX on US confirmed on upright CXR
3	Trauma ward	Office	True negative	True negative	
4	ICU	Office	True negative	True negative	Well-defined B-lines increased confidence in excluding PTX
5	Neurosurgery ward	Home	True negative	True negative	
6	Neurosurgery ward	Office	True negative	True negative	
7	Trauma ward	Hotel, UK	False negative	True negative	Trans-Atlantic case reference standard result still uncertain
8	Trauma ward	Office	True positive	True negative	Subcutaneous emphysema clearly noted and determined abnormal
9	Trauma ward	Office	True negative	True negative	
10	Trauma ward	Office	True negative	True positive	Final impression was sub-Q emphysema
11	Trauma ward	Office	True negative	True negative	First case with M-mode capability
12	Trauma ward	Office	True negative	True negative	
13	Trauma ward	Office	True negative	True positive	Bedside nurse was guided to make diagnosis the first time she ever held the ultrasound probe

### Statistical analysis

Ultrasound findings on all lung fields were tabulated as positive or negative for PTXs and compared to the subsequent upright CXR report as the reference standard. Dichotomous data were analyzed using Stata version 12.0 (Stata Corp., College Station, TX, USA) by calculating rates of true positives, true negatives, sensitivity, and specificity.

## Results

### Quantitative results

Thirteen patients (26 lung fields) were examined. The remote exam was possible in all cases with ultimately good connectivity including one trans-Atlantic interpretation (Table 1). Compared to the subsequent upright CXR report as the perceived reference standard, there were 4 true-positive remotely diagnosed PTXs, 2 false-negative diagnoses, and 20 true-negative diagnoses for 66% sensitivity (95% confidence interval (CI) 0.22 to 0.96), 100% specificity (95% CI 0.59 to 1.00), 100% positive predictive value (95% CI 0.40 to 0.97), and 92% accuracy for remotely guided chest examination. The likelihood ratio of a positive test was infinite, and the likelihood ratio of a negative test was 0.33 (95% CI 0.11 to 1.03). No patient, including those diagnosed with a post-TT removal PTX, required re-insertion of a pleural drain.

### Illustrative cases

**Figure 2:**
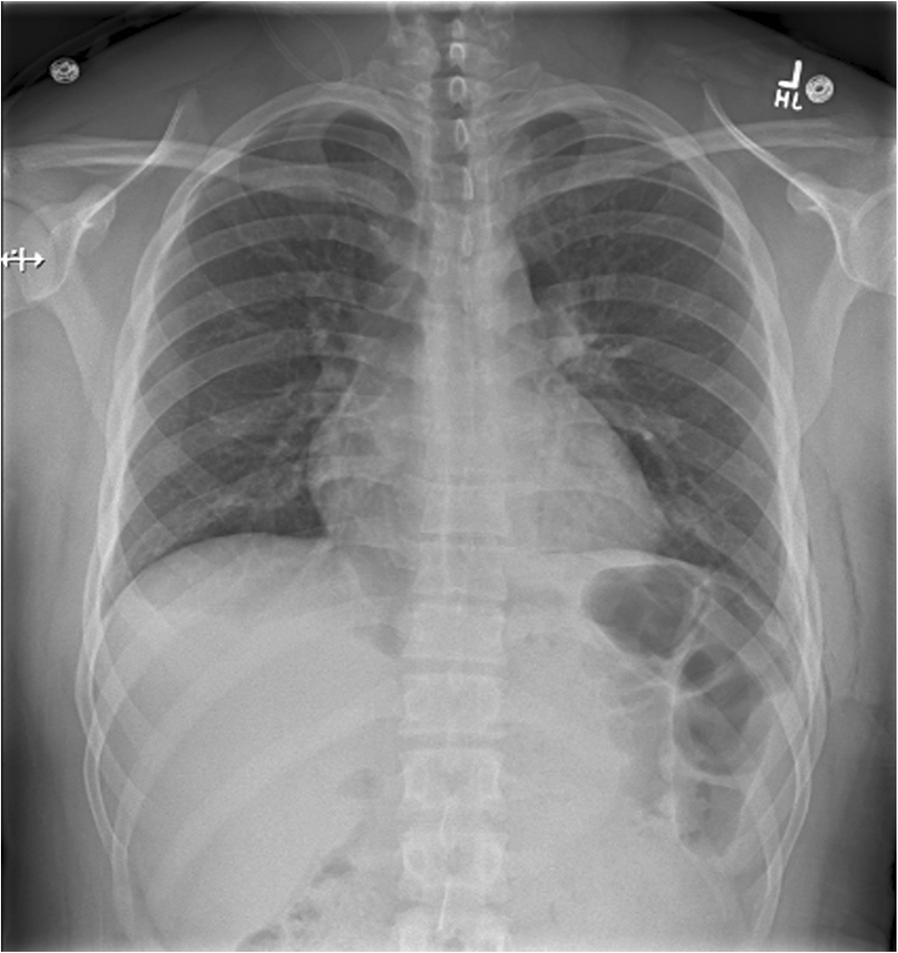
**Case 1 in which the final radiology report noted a ‘tiny residual left apical pneumothorax.’** This pneumothorax was not detected after concluding the RTMUS exam.

**Figure 3:**
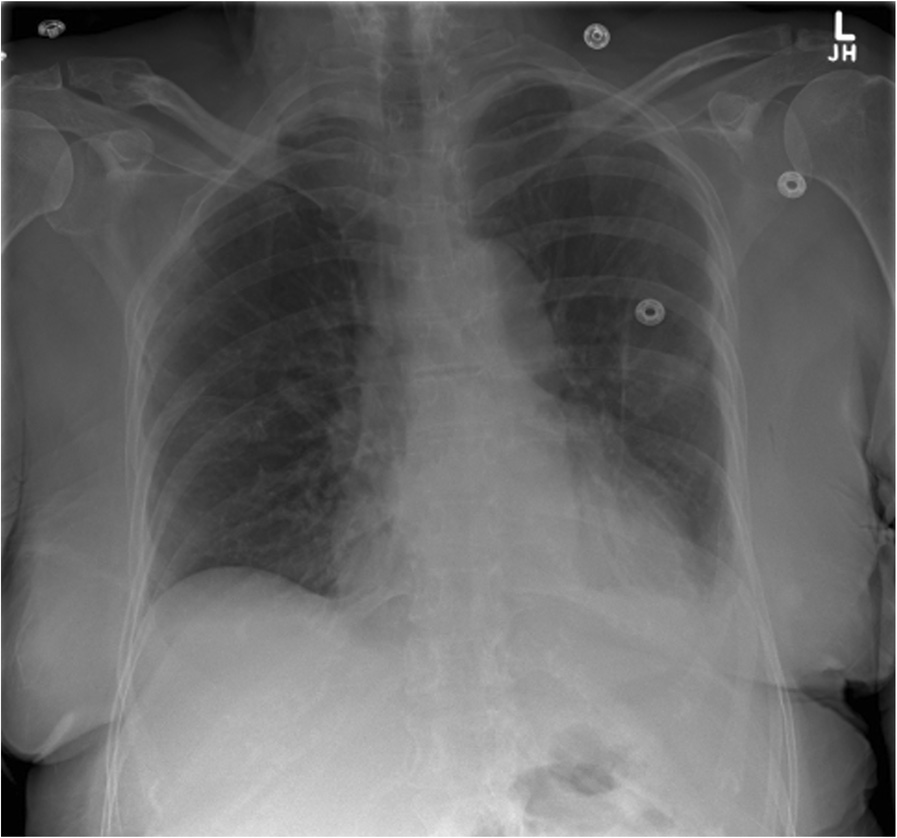
**Case 7: chest radiograph obtained after removal of a left-sided tube thoracostomy.** The chest radiograph noted a ‘small focal lucency at the right apex that could represent a small loculated pneumothorax.’

**Figure 4:**
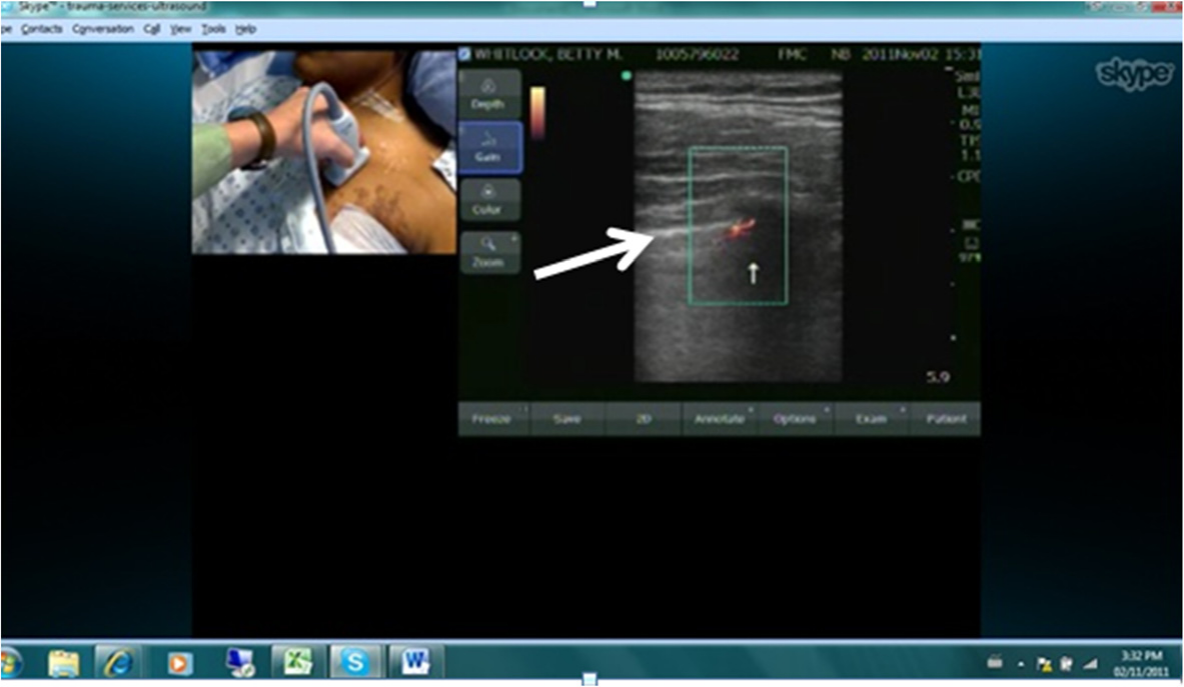
**Case 1: remote mentor's computer screen.** The screen demonstrated the nurse practitioner's placement of the ultrasound probe and the resultant ultrasound image that depicted a color power Doppler signal from the pleural interface, suggesting the presence of lung sliding at this anatomic location. The large white arrow designated the parietal-visceral pleural interface, and the small white arrow indicated the color power Doppler signal seen at this interface.

**Figure 5:**
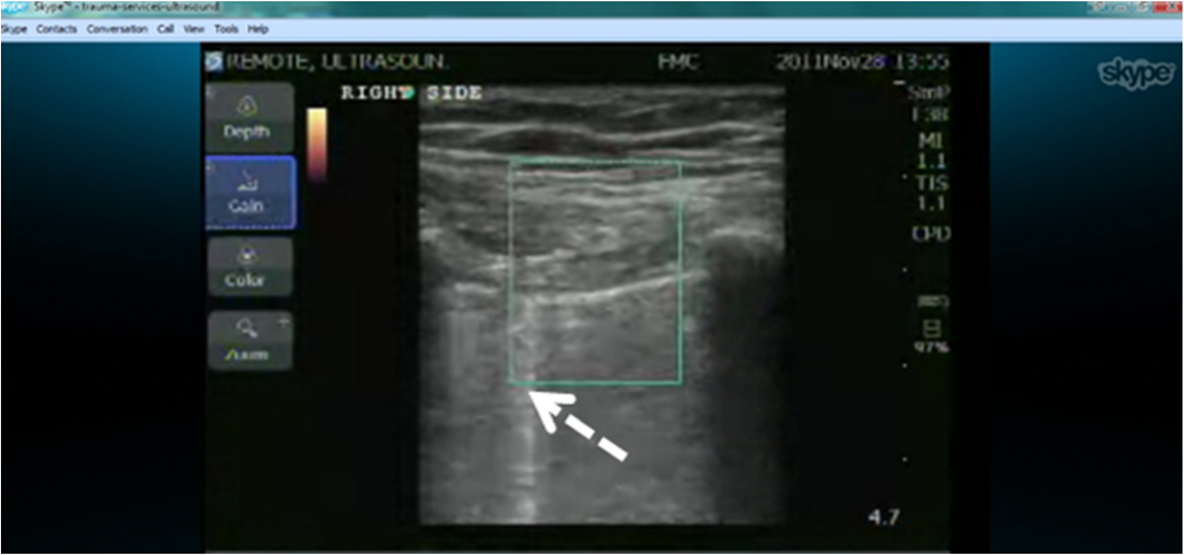
**Case 7: screen capture of mentor's screen in England.** The screen demonstrated the image generated in Calgary suggesting a visceral-parietal pleural interface without an obvious power-slide, but a comet-tail artifact (B-line) (dashed arrow) emanating from the pleural interface.

Specific cases are illustrative and novel, constituting the first reported examples of these techniques to our knowledge. There were two false-negative examinations. In case 1, the final radiology report noted ‘a tiny residual left apical pneumothorax’ on CXR (Figure 2). In case 7 which was conducted across the Atlantic Ocean, the final CXR report was of ‘a small focal lucency noted at the right apex that could represent a small loculated pneumothorax,’ noting this was opposite from the side of TT removal (Figure 3). In both these cases, the remote expert did not appreciate a PTX as there appeared to be pleural apposition and lung sliding (Figures 4 and 5). Case 7 involved the greatest geographic separation with the examiner and patient being in Calgary, while the mentor was online in Heathrow, England (7,058 km distant). In this case, obvious lung sliding was not apparent, but the final clinical call was ‘no true sliding seen but comet-tails and lung pulse - designated No PTX,’ suggesting a possible error in interpretation rather than remote imaging accuracy.

**Figure 6:**
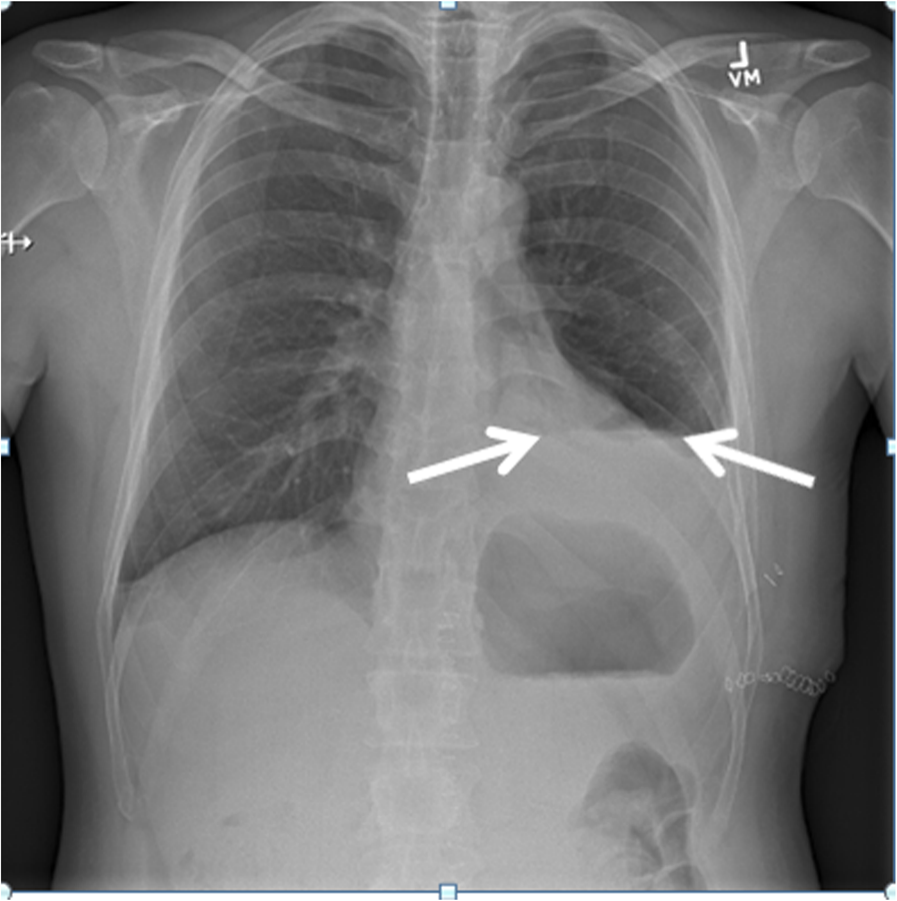
**Case 2: left-sided hydropneumothorax after tube thoracostomy removal with a reported maximal diameter of 15 mm.** Dual arrows indicate air-fluid level of hydropneumothorax.

There were also four true-positive examinations, in which abnormalities of pleural sliding were correctly diagnosed, corresponding to four reported post-TT removal CXRs with a 15-mm pleural separation (Figure 6; case 2), a trace PTX with 5 to 6 mm of separation (case 8), an 8-mm pleural separation (case 10), and a ‘tiny’ PTX (case 13). The last diagnosis constituted a remarkable case in which a registered nurse who had no prior ultrasound experience and had never picked up an ultrasound probe previously was mentored to detect the subtle and tiny PTX which was denoted by clear delineation of a lung point at the lung apex. This single case has thus been reported separately [15]. The lack of sliding was confirmed both visually and through M-mode interrogation. There appeared to be a persistent junction between areas where the lung was clearly sliding with respiration and not on either side of the hyperlucent pleural line interpreted to represent a small focus of intrapleural air, which is by definition a loculated PTX. This description of a localized small PTX has recently been corroborated and termed the ‘double lung point.’

## Discussion

RTMUS is a technique that permits willing but inexperienced personnel to perform accurate POC ultrasound examinations on behalf of a remote expert. The critical requirement to enable this is Internet connectivity and a willing remote expert, as the technical requirements are otherwise modest. With this concept, nurses can examine the pleural spaces to detect post-TT removal pneumothoraces. However, many other forms of POC ultrasound examinations could also be performed as dictated by the clinical situation.

Lung ultrasound was chosen as the basis of this study as it is rapidly becoming a critically important technique in acute care situations and is applicable to almost any situation that concerns respiratory function [10,16]. With experience, the technique is more accurate than supine CXR, and the physical size of PTXs can be demonstrated according to chest topography [17].

It remains commonplace in many institutions, including our own, to obtain routine CXRs after TT removal, the utility and cost-effectiveness of which is uncertain and debated [18,19]. As there are downsides such as cost, patient transport requirements, and radiation, it is attractive to consider ultrasound which can be performed at the bedside. Limited experience in other institutions concerns lung ultrasound in the management of TTs. Goudie et al. [20] performed a mean of 3.0 ultrasound examinations whenever a CXR was obtained post-operatively after thoracic surgery and noted a sensitivity of only 21% for the ultrasound detection of PTXs in a cohort of 120 patients. Dente et al. followed 14 patients with TTs, performing a median of 7 exams and noting that while the sensitivity for ultrasound was 100% in the first 24 h after TT placement, it fell to 55% after 24 h with TT in place, which they suggested was the result of induced pleural adhesions [21]. This contrasts with Saucier et al. who noted perfect agreement (kappa 1.0) between ultrasound and CXR after TT removal in 50 patients [22]. This improved performance might be explained by the experience of the primary ultrasonographer who performed 98% of the examinations [22].

Evidence from these studies suggests that a significant learning curve or critical level of experience is required to detect PTX using lung ultrasound after TT removal. We believe, therefore, that bringing increased experience to the bedside with RTMUS and which permits utilization of advanced ultrasound techniques is a key to accuracy. In this pilot study, the exam had excellent test performance with an infinite likelihood ratio of a positive test. For analytic purposes, two cases in which reportedly tiny PTXs were apparent on a subsequent CXR were considered false-negative examinations from the perspective of PTX detection. However, no patient required TT re-insertion, and all the recurrent PTXs were managed conservatively. Thus, if the purely clinical perspective is taken, regarding solely the need to detect large PTXs requiring intervention, there were no such cases missed, and the detection of lung sliding at the lung apices was reassuring that no large PTX was present in these patients. Until further experience is gained, we would therefore suggest that a possible paradigm for the introduction of mentored post-TT removal lung examination is that the ultrasound be performed soon after TT removal to ensure that no large air leak has occurred. If this examination is reassuring, then a chest radiograph can be obtained in a routine fashion unless the clinical status changes. It is not unlikely that in the future, radiographs may be obviated by greater use of ultrasound either remotely mentored or simply as all clinicians gain experience and accuracy.

A limitation of the study is the small sample size. Another limitation was the use of the chest radiograph as the reference standard test. While upright CXRs perform better than supine, this modality still constitutes a two-dimensional examination for a three-dimensional problem. For instance, the actual truth regarding the presence or not of a PTX for case 7 will never truly be known. A further technical limitation was with the ultrasound machine used in the study which initially did not have an M-mode or video capture capability. Late in the cohort, a software upgrade did provide M-mode capability, and this feature of the examination was utilized in the last two cases where this technique may have enabled a remarkable diagnosis of a subtle PTX by an ultrasound-naive nurse using ultrasound for the first time ever (case 13). The main technological barrier encountered was the connection reliability and troubleshooting of the off-the-shelf components. On several occasions, the RTMUS did not immediately connect the mentor with the nurse at the bedside when desired, but all connectivity and configuration issues were resolved through the involved clinical personnel without input from professional informatics specialists. Ultimately, however, what is technically hard today becomes easy in the future as the world connects electronically and devices improve.

## Conclusions

In summary, marrying advances in ultrasound technology with simple and available informatics technologies permits experienced ultrasonographers to direct remote ultrasound examinations wherever Internet connectivity is available. Thus, with organization and commitment, a broadly experienced clinically orientated ultrasound mentor could support multiple clinical providers in multiple locations even if they were geographically dispersed on a worldwide basis. Ongoing study will now be required to assess the impact and guide the integration of this approach into contemporary practice.

## Abbreviations

CODEC: Compression or decompression for digital video; CXR: Chest X-ray radiograph; GUI: Graphical user interface; ICU: Intensive care unit; LAN: Local area network; POC-US: Point-of-care ultrasound; PTX: Pneumothorax; RTMUS: Remote telementored ultrasound; TT: Tube thoracostomy; USB: Universal serial bus; VOIP: Voice over internet protocol.

## Competing interests

The authors declare that they have no competing interests.

## Authors’ contributions

NB designed the study, collected and analyzed the data, and critically reviewed the manuscript. PBM did the literature search, designed the study, analyzed and interpreted the data, and critically reviewed the manuscript. CT analyzed and interpreted the data and critically reviewed the manuscript. DRH did the literature search, interpreted the data and critically reviewed the manuscript. ZX analyzed and interpreted the data and critically reviewed the manuscript. IC did the literature search, designed the study, interpreted the data, and critically reviewed the manuscript. MTM collected the data and critically reviewed the manuscript. NM designed the study and wrote and critically reviewed the manuscript. CGB designed the study, interpreted the data, and critically reviewed the manuscript. LP interpreted the data and critically reviewed the manuscript. AWK did the literature search, designed the study; collected, analyzed, and interpreted the data; and wrote and critically reviewed the manuscript. All authors read and approved the final manuscript.
